# Metabolomics profiles associated with diabetic retinopathy in type 2 diabetes patients

**DOI:** 10.1371/journal.pone.0241365

**Published:** 2020-10-29

**Authors:** Jun Ho Yun, Jeong-Min Kim, Hyun Jeong Jeon, Taekeun Oh, Hyung Jin Choi, Bong-Jo Kim

**Affiliations:** 1 Division of Genome Research, Center for Genome Science, Korea National Institute of Health, Cheongju, Chungbuk, Republic of Korea; 2 College of Pharmacy, Chungbuk National University, Cheongju, Chungbuk, Republic of Korea; 3 Department of Internal Medicine, Chungbuk National University College of Medicine, Cheongju, Chungbuk, Republic of Korea; 4 Department of Biomedical Sciences & Department of Anatomy and Cell Biology, Wide River Institute of Immunology, Seoul National University College of Medicine, Seoul, Republic of Korea; Children's Hospital Boston, UNITED STATES

## Abstract

Diabetic retinopathy (DR) is a common complication of diabetes, and it is the consequence of microvascular retinal changes due to high glucose levels over a long time. Metabolomics profiling is a rapidly evolving method used to identify the metabolites in biological fluids and investigate disease progression. In this study, we used a targeted metabolomics approach to quantify the serum metabolites in type 2 diabetes (T2D) patients. Diabetes patients were divided into three groups based on the status of their complications: non-DR (NDR, n = 143), non-proliferative DR (NPDR, n = 123), and proliferative DR (PDR, n = 51) groups. Multiple logistic regression analysis and multiple testing corrections were performed to identify the significant differences in the metabolomics profiles of the different analysis groups. The concentrations of 62 metabolites of the NDR versus DR group, 53 metabolites of the NDR versus NPDR group, and 30 metabolites of the NDR versus PDR group were found to be significantly different. Finally, sixteen metabolites were selected as specific metabolites common to NPDR and PDR. Among them, three metabolites including total DMA, tryptophan, and kynurenine were potential makers of DR progression in T2D patients. Additionally, several metabolites such as carnitines, several amino acids, and phosphatidylcholines also showed a marker potential. The metabolite signatures identified in this study will provide insight into the mechanisms underlying DR development and progression in T2D patients in future studies.

## Introduction

Diabetic retinopathy (DR), like diabetic neuropathy and nephropathy, is a common complication of diabetes. It is the leading cause of loss of vision in diabetic patients. Long-standing disease, along with hyperglycemia, hyperlipidemia, hypertension, and genetic factors, is a major risk factor of diabetes retinopathy [1–3]. DR can be classified into non-proliferative DR (NPDR) and proliferative DR (PDR) depending on the presence of neovascularization. NPDR is the early retinal disease without neovascularization, while PDR is the more progressed form of NPDR with the development and growth of new variable-sized vessels on the retinal surface [4–6].

Quantitative analyses of small-molecule metabolites in biological specimens such as blood and urine can be performed due to the rapid advances in metabolomics. It is well known that metabolic phenotype is the end product of the interaction between genetic and environmental factors, and it reflects the pathophysiological conditions of various diseases. Therefore, the association of these metabolites with biological processes must be studied. Several large-scale metabolomics profiling studies have been performed to identify the metabolites related to disease progression [7–9]. However, there are a limited number of large-scale metabolomics profiling studies on the blood metabolites associated with DR. To date, several metabolites have been identified as markers of DR, and their related metabolism pathways have been elucidated [10–12]. Among these metabolites, plasma asymmetric dimethylarginine (ADMA) was found to be associated with the early changes in retinal vessels in DR and the final status of DR progression [12–14]. Recently, four metabolites, fumaric acid, uridine, acetic acid, and cytidine, have been reported as candidate biomarkers of PDR progression [15].

Some studies have identified blood metabolites associated with DR in patients who may or may not have received drug treatment. However, it can be speculated that these identified metabolites may vary depending on the study cohort and analysis methods. Therefore, identifying additional metabolites that could be useful biomarkers of DR progression and potential targets for early treatment and prevention of diabetic complications will be beneficial.

Thus, in this study, we obtained and analyzed the metabolome data of DR patients using a targeted metabolomics approach and identified several metabolites that showed differences in their concentrations in non-DR (NDR), NPDR, and PDR type 2 diabetes (T2D) patients.

## Materials and methods

### The study population and samples

Totally, 317 T2D patients [143 NDR patients, 123 NPDR patients, and 51 PDR patients] at Chungbuk National University Hospital were included in this study. DR was diagnosed by dilated fundus examination performed by a retina specialist. Gender, age, height, weight, body mass index (BMI), and HbA1c, glucose, and creatinine levels of all patients were recorded. Serum samples of the T2D patients with or without DR were collected and stored at -80°C in a refrigerator, according to the international ethical guidelines. All patients provided written informed consent. This study was reviewed and approved by the Chungbuk National University Hospital Institutional Review Board (Approval Number: CBNUH 2015-02-006).

### Metabolomics profiling

The serum samples of the T2D patients were analyzed using a targeted metabolomics approach. To quantify the metabolites, liquid chromatography (LC) and flow-injection analysis (FIA)–mass spectrometry (MS) were performed using the AbsoluteIDQ^®^ p180 Kit (BIOCRATES Life Sciences AG, Innsbruck, Austria), and quality control was performed to select metabolites for further analyses. Metabolites with significant differences in their concentrations in the different analysis groups were identified. All procedures were performed as described previously [16, 17]. Briefly, the serum samples were analyzed using the API 4000 QTRAP LC/MS/MS system (Applied Biosystems, Foster City, CA, USA) and the Agilent 1200 HPLC system (Agilent Technologies, Santa Clara, CA, USA), following the manufacturers’ instructions. Calibration standards and three different quality controls were established using the AbsoluteIDQ^®^ p180 Kit to serve as references for calculating the metabolite concentrations. Two additional pooled normal human serum samples in each plate were used as reference standards. Data quality of each metabolite was checked according to the following criteria. First, the coefficient of variation of the metabolites in 10 reference standards must be below 15%. Second, 50% of the measured metabolite concentrations in both the reference and experimental samples must be above the limit of detection, which was set to three times the median value of the three blank samples in each plate. After the quality control processes, 122 metabolites were found to meet the above-mentioned criteria, and they were thus selected for further statistical analyses.

### Statistical analyses

**Fig 1:**
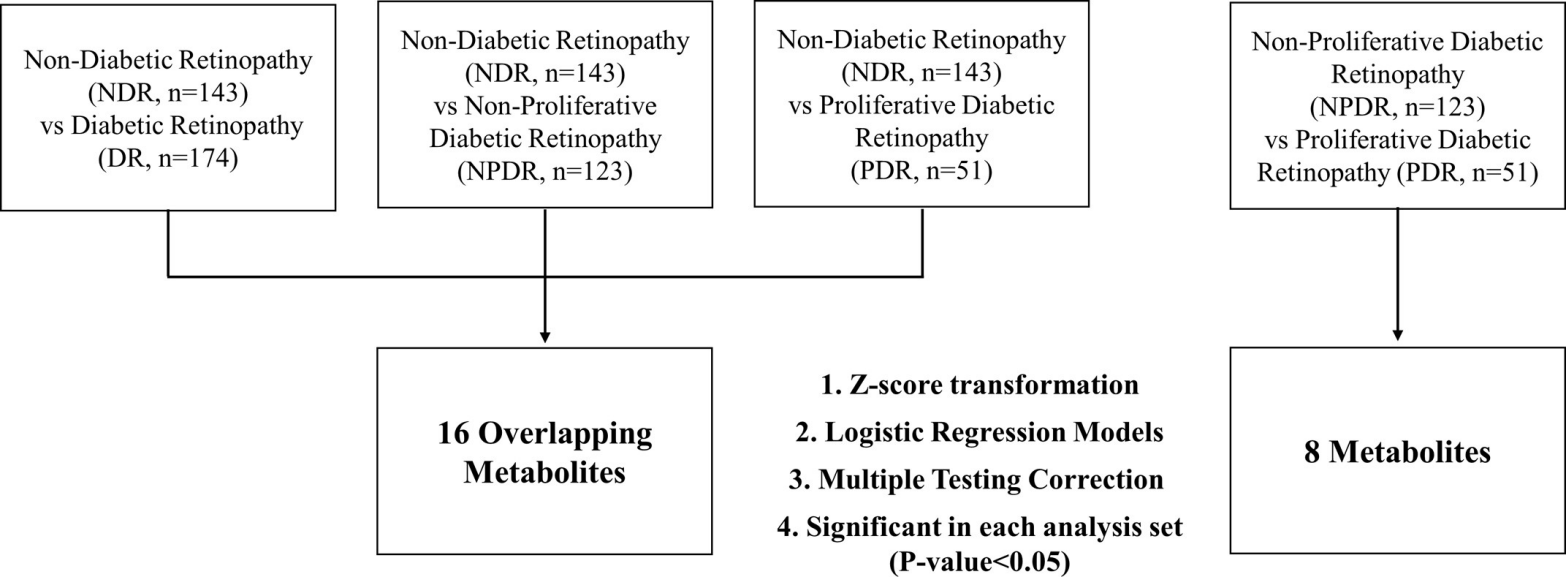
An overview of the metabolomics analysis workflow.

Statistical analyses were performed using the R software (version 3.6.2; http://www.r-project.org). To identify the metabolites with different concentrations in the serum of NDR, NPDR, and PDR patients, the odds ratio (OR) with a 95% confidence interval (CI) (per metabolite) was calculated using a multiple logistic regression model to adjust for covariates such as age or gender. To obtain a mean of zero and a standard deviation of one, the metabolite concentrations were log-transformed and normalized. To adjust the p-value for multiple comparisons, the false discovery rate (FDR) was calculated using the p.adjust function in the R ‘Stats’ package (FDR < 0.05) (Fig 1). To confirm the result of the logistic regression analysis, the ANCOVA analysis, included in the R package, was performed to calculate fold change values.

## Results

### Clinical and biochemical characteristics of the study population

The study population was composed of T2D patients (n = 317), including NDR (n = 143) and DR (n = 174) patients (Table 1). DR patients were further divided into two groups based on the status of the complications. These included the NPDR (n = 123) and PDR (n = 51) groups. Average T2D duration of each group was 7.1 years (NDR), 14.9 years (NPDR), and 20 years (PDR). HbA1c and creatinine concentrations in the three analysis groups were found to be significantly different. However, glucose levels and age of two analysis groups NDR versus DR and NDR versus NPDR, but not of NDR versus PDR, were found to be significantly different. Gender of only the NDR versus PDR analysis group was found to be significantly different. There were no significant differences in the height, weight, and BMI of the three analysis groups. Age was used as a covariate for NDR versus DR and NDR versus NPDR analyses, while gender was used as a covariate for NDR versus PDR analysis.

**Table 1:** Clinical and biochemical characteristics of the study population.

Clinical and biochemical parameters	NDR (n = 143)	DR (n = 174)	*p-value (NDR versus DR)*	NPDR (n = 123)	*p-value (NDR versus NPDR)*	PDR (n = 51)	*p-value (NDR versus PDR)*	*p-value (NPDR versus PDR)*
Female (%)	37.8	41.9	0.4494	39.8	0.73	47.1	< 0.01	0.3885
Age (years)	54.89 (11.34)	62.18 (11.66)	< 0.01	62.60 (11.60)	< 0.01	61.18 (11.87)	0.257	0.4698
Height (cm)	164.29 (8.58)	162.95 (8.59)	0.1878	162.94 (9.14)	62.48 (10.89)	162.97 (7.22)	0.3061	0.9809
Weight (kg)	66 (10.76)	65.95 (10.12)	0.5602	66.03 (10.43)	164.03 (7.09)	64.99 (9.38)	0.3771	0.5272
BMI (kg/m^2^)	24.53 (3.38)	24.76 (3.41)	0.5606	24.89 (3.56)	67.17 (8.21)	24.48 (3.06)	0.919	0.6432
HbA1c (%)	7.38 (1.84)	8.19 (1.89)	< 0.01	8.07 (1.78)	< 0.01	8.49 (2.14)	< 0.01	0.2363
Glucose (mg/dL)	148.20 (59.58)	173.52 (77.41)	< 0.01	172.98 (71.65)	< 0.01	174.82 (90.61)	0.05955	0.8999
Creatinine (mg/dL)	0.96 (0.18)	1.21 (0.58)	< 0.01	1.10 (0.36)	< 0.01	1.49 (0.87)	< 0.01	< 0.01
T2D duration (years)	7.1 (4.9)	16.43 (11.1)	-	14.9 (8.2)	-	20 (15.48)	-	-

* DR, diabetic retinopathy; NDR, non-diabetic retinopathy; NPDR, non-proliferative diabetic retinopathy; PDR, proliferative diabetic retinopathy; BMI, body mass index; T2D, type 2 diabetes.

### Identification of the metabolites with differences in their concentrations in DR and NDR patients

To identify metabolites with significant differences in their concentrations in the DR and NDR groups, a multiple logistic regression analysis was performed using DR as a dependent variable and each metabolite as an explanatory variable, with adjustment for age. The concentrations of 62 metabolites in the two groups differed significantly (*P* < 0.05) (S1 Table). Among them, several metabolites, such as two acylcarnitines (propionylcarnitine [C3] and butyrylcarnitine [C4]), one amino acid (proline), two biogenic amines (creatinine and total dimethylarginine [DMA]), and hexose, showed significantly higher concentrations in the DR group than in the NDR group.

### Identification of the common metabolites with differences in their concentrations in the NPDR and NPR patients, compared to the NDR patients

**Fig 2:**
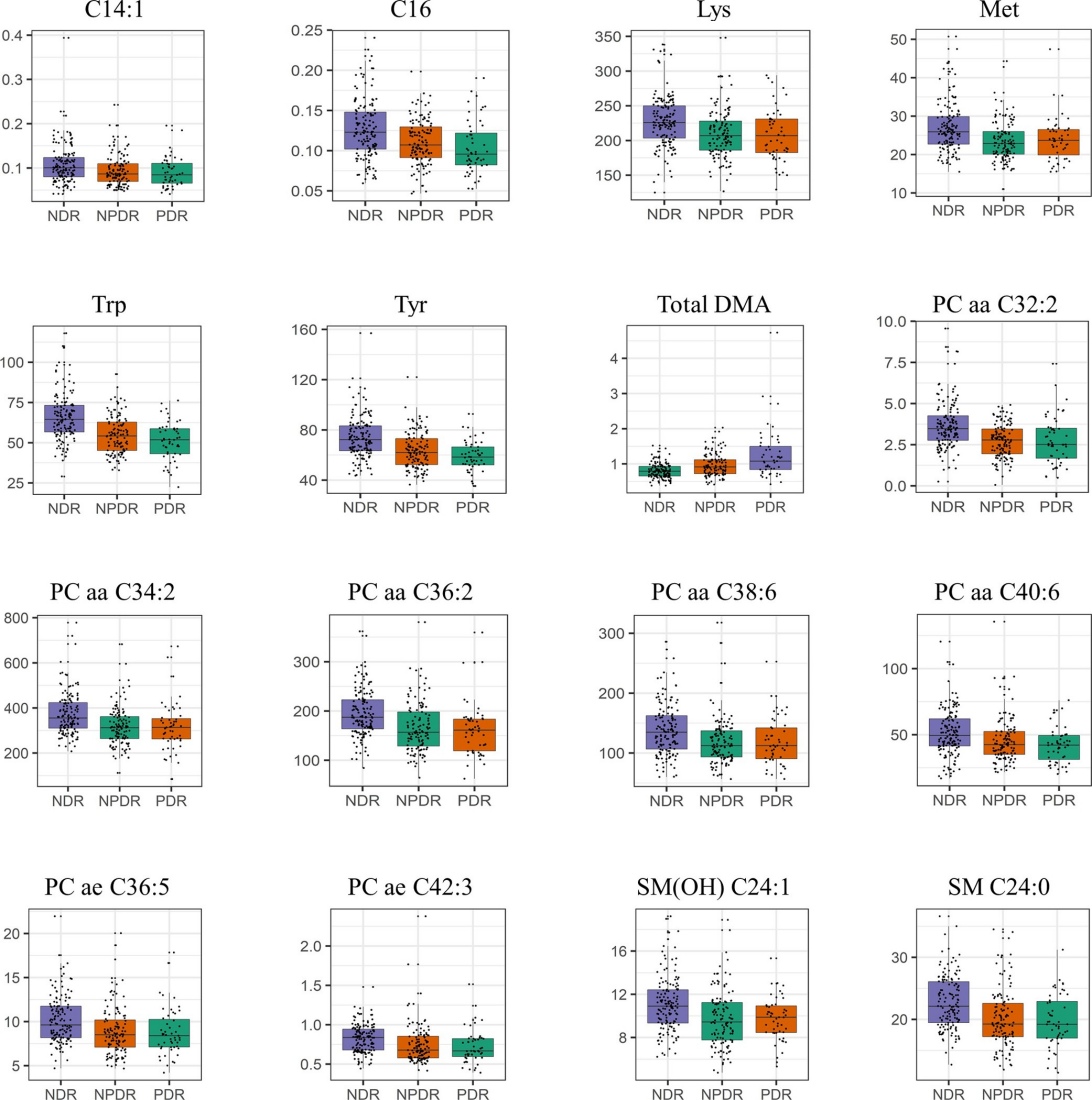
The levels of 16 DR-specific metabolites. Dot- and box- plots represent the concentrations of the relevant metabolites in the serum of type 2 diabetes patients with non-diabetic retinopathy (NDR), non-proliferative DR (NPDR), and proliferative diabetic retinopathy (PDR). Graphs were plotted using the R package (Stats, version 3.6.2). The Y-axis indicates the metabolite concentration in micromoles (μM). P-values were determined via ANCOVA analysis. Fourteen metabolites (except PC ae C36.5 and PC ae 42.3) showed p-values lower than 0.001. Abbreviations: C:14:1, tetradecenoylcarnitine; C:16, hexadecanoylcarnitine; DMA, dimethylarginine; PC aa, phosphatidylcholine diacyl; PC ae, phosphatidylcholine acyl-alkyl; SM, sphingomyelin.

To identify the common metabolites with significant differences in their concentrations in the two analysis groups, NDR versus NPDR and NDR versus PDR, the same statistical analysis was performed for each group. Then, the common metabolites of the groups in each analysis group were selected. Fifty-three metabolites showed significantly different concentrations in the NDR and NPDR groups (S2 Table). Three amino acids (alanine, aspartic acid, and glutamine), total DMA, and hexose showed higher concentrations in the NPDR patients than in the NDR patients. Three acylcarnitines (carnitine [C0], tetradecenoylcarnitine [C14:1], and hexadecanoylcarnitine [C16]), seven amino acids (arginine, histidine, lysine, methionine, threonine, tryptophan, and tyrosine), and thirty-eight glycerophospholipids showed lower concentrations in the NPDR patients than in the NDR patients. Thirty metabolites showed significantly different concentrations in the PDR compared to NDR patients (S3 Table). Three short-chain acylcarnitines (C3, C4, and valerylcarnitine [C5]) and three biogenic amines (creatinine, kynurenine, and total DMA) showed significantly higher concentrations in the PDR patients than in the NDR patients. On the other hand, five long-chain acylcarnitines (C14:1, C16, octadecanoylcarnitine [C18], octadecenoylcarnitine [C18:1], and octadecadienylcarnitine [C18:2]), thirteen glycerophospholipids, and five amino acids (lysine, methionine, serine, tryptophan, and tyrosine) showed lower concentrations in the PDR patients than in the NDR patients. Sixteen common metabolites with significantly different concentrations in both the NPDR and PDR patients, compared to the NDR patients, were finally selected as specific metabolites (Fig 2 and Table 2). Among them, only total DMA showed significantly higher concentrations in the PDR and NPDR patients than in the NDR patients, while the other metabolites showed lower concentrations in the PDR and NPDR patients, compared to the NDR patients.

**Table 2:** Identification of the common metabolites associated with both non-proliferative and proliferative diabetic retinopathy.

Metabolites	Logistic regression		ANCOVA	
	Odds Ratio (95% CI)	*p-value* (FDR corrected)	Fold Change	*p-value*
Tetradecenoylcarnitine (C14:1)	0.63 (0.48–0.82)	4.32E-03	0.87	3.51E-04
Hexadecanoylcarnitine (C16)	0.59 (0.45–0.75)	6.68E-04	0.86	1.95E-05
Lysine (Lys)	0.63 (0.49–0.81)	2.90E-03	0.92	2.15E-04
Methionine (Met)	0.53 (0.4–0.69)	1.69E-04	0.88	9.26E-07
Tryptophan (Trp)	0.36 (0.26–0.49)	4.48E-08	0.81	1.79E-12
Tyrosine (Tyr)	0.43 (0.31–0.57)	1.42E-06	0.84	4.84E-10
Total Dimethyarginine (Total DMA)	2.3 (1.59–3.47)	4.28E-04	1.31	4.73E-05
Phosphatidylcholine diacyl C32:2 (PC aa C32:2)	0.47 (0.34–0.62)	1.82E-05	0.75	4.79E-08
Phosphatidylcholine diacyl C34:2 (PC aa C34:2)	0.56 (0.42–0.73)	6.68E-04	0.85	2.24E-05
Phosphatidylcholine diacyl C36:2 (PC aa C36:2)	0.56 (0.43–0.73)	4.28E-04	0.85	1.10E-05
Phosphatidylcholine diacyl C38:6 (PC aa C38:6)	0.61 (0.47–0.78)	1.49E-03	0.86	8.06E-05
Phosphatidylcholine diacyl C40:6 (PC aa C40:6)	0.6 (0.46–0.77)	1.15E-03	0.86	5.91E-05
Phosphatidylcholine acyl-alkyl C36:5 (PC ae C36:5)	0.67 (0.51–0.86)	1.03E-02	0.87	1.46E-03
Phosphatidylcholine acyl-alkyl C42:3 (PC ae C42:3)	0.69 (0.53–0.88)	1.60E-02	0.9	2.30E-03
Hydroxysphingomyeline C22:1 (SM (OH) C22:1)	0.6 (0.46–0.77)	1.22E-03	0.88	6.09E-05
Sphingomyeline C24:0 (SM C24:0)	0.57 (0.44–0.74)	4.28E-04	0.88	7.84E-06

### Identification of the metabolites with differences in their concentrations in the NPDR and PDR patients

Eight metabolites with significantly different concentrations in PDR and NPDR patients were selected (S4 Table). Among them, four metabolites (pimelycarnitine [C7:DC], creatinine, total DMA, and phosphatidylcholine [PC] aa C32:2) showed higher concentrations in the PDR patients than in the NPDR patients. The other four metabolites (lyso PC a C18:2, PC aa C36:1, PC aa C44:4, and PC aa C44:5) showed lower concentrations in the PDR patients than in the NPDR patients.

## Discussion

Using a high-throughput targeted metabolomics strategy, metabolites associated with DR in T2D patients were determined. Sixteen metabolites, including total DMA, were identified as common indicative metabolites that showed different concentrations in both the PDR and NPDR patients, compared to the NDR patients. Of note, among them, total DMA showed higher levels in the serum of both NPDR and PDR patients than those in NDR patients. The concentrations of the other fifteen metabolites, including two acylcarnitines (C14:1 and C16), four amino acids (lysine, methionine, tryptophan, and tyrosine), seven glycerophospholipids, and two sphingomyelins, were lower in both the NPDR and PDR patients than in the NDR patients. Therefore, these metabolites might be linked to diabetic complications, particularly DR, in T2D patients.

Total DMA is the sum of ADMA and symmetric DMA (SDMA). ADMA was reported to inhibit nitric oxide synthase (NOS), resulting in a lack of nitric oxide (NO). This, in turn, led to the development of diabetic complications, suggesting the association between diabetic complications and ADMA to some extent. Therefore, it is reported that plasma ADMA levels are associated with retinopathy in T2D patients [12, 18, 19]. In this study, the concentration of total DMA was significantly higher (OR = 2.3) in both PDR and NPDR patients, compared with NDR patients (Table 2). This result was consistent with those of previous studies on the association of ADMA with NO in DR and even on the association of increased ADMA concentrations with cardiovascular risk [12, 20, 21]. NO shortage is known to accelerate DR by increasing the generation of oxygen and nitrogen reactive species [10, 12]. Therefore, it was also suggested that elevated circulating plasma ADMA levels were associated with DR in T2D. Based on the results of previous studies and of our own, total DMA and ADMA may be indicative metabolites of DR, associated with the regulation of NO synthesis in the blood of T2D patients.

Tryptophan is metabolized to kynurenine by indoleamine 2,2-dioxygenase (IDO). There are several reports on the tryptophan-kynurenine pathway in several diseases; however, the reports of this pathway in the context of diabetic retinopathy are rare. For instance, it was reported that the altered tryptophan-kynurenine metabolism pathway was associated with diabetic cataracts and several human diseases related to nervous and immune systems [22, 23]. Recently, the role of the tryptophan-kynurenine metabolism pathway has been studied in animal models and human individuals with retinal and optic nerve damage, but not diabetic retinopathy. Of note, tryptophan was metabolized to kynurenine, and its concentration was decreased in the abovementioned models. The concentration of kynurenine, the product of tryptophan metabolism, was reciprocally increased [24]. It was reported that a higher IDO expression was observed in NPDR patients than in NDR patients; interestingly, an even higher IDO expression was observed in PDR versus NPDR patients. Due to higher IDO expression, kynurenine levels were also increased in NPDR and PDR versus NDR patients. However, no significant change in the levels of tryptophan was observed. Of note, the authors analyzed the concentration of only four metabolites related to kynurenine and tryptophan and measured the expression of IDO using a total of 81 samples collected from 35 control subjects, 22 NPDR patients, and 24 PDR. [25]. On the other hand, here, we analyzed the concentration of 180 metabolites including tryptophan and kynurenine, simultaneously, and in a much higher number of patients (requiring only a small sample volume since we used a high-throughput method); of note, we identified three known, and thirteen unknown metabolites related to DR (Figs 1 and 2). In our study, the levels of tryptophan were decreased in both NPDR and PDR patients, compared to those in NDR patients (Table 2). Furthermore, the levels of kynurenine were significantly higher in PDR versus NDR patients (OR 1.75) (S3 Table and S1 Fig). Of note, they were also higher, in PDR versus NPDR patients, and in DR versus NDR patients; however, these differences were not statistically significant (S5 Table and S1 Fig). Altogether, the results of our study further support the exploration of tryptophan and kynurenine for the treatment of DR.

It has been reported that serum creatinine is an indicative marker of renal dysfunction, which is also induced by long-standing diabetes. Furthermore, a higher serum creatinine level was found to be associated with PDR and diabetic nephropathy (DN) development [26]. In our study, creatinine level was significantly higher in the PDR patients than in the NDR and NPDR patients, and this finding was consistent with the clinical characteristics of the study population, as shown in Table 1. As a result, the creatinine level was significantly higher in the DR patients than in the NDR patients (S1–S4 Tables). DR, like DN, is one of the most common microvascular complications of diabetes. Renal dysfunction is associated with DR development and deterioration [27]. Additionally, it has been reported that DR is a prognostic factor of progression of chronic kidney disease in T2D patients [9]. These results could suggest that serum creatinine is also a candidate marker of DR development in diabetic patients.

Due to the difficulty in obtaining vitreous samples from human patients, blood plasma and serum samples were obtained for metabolomics profiling of DR. To date, very few studies have reported the metabolites associated with DR using large-scale diabetic patient sample profiling [10]. Chen et al. have reported that signatures of several metabolites, such as 2-deoxyribonic acid, 3,4-dihydroxybutyric acid, erythritol, gluconic acid, and ribose, were associated with DR progression in diabetic patients [11]. However, none of these metabolites was found in our study probably due to the use of a different analysis platform. In another recent study, two metabolites, arginine and carnitine, were found to be altered in DR patients, compared to diabetic controls. Arginine levels were significantly higher in the DR patients than in the diabetic controls, and carnitine concentration was elevated in the PDR patients compared to the NPDR patients [1]. However, in our study, arginine concentration was not significantly changed in the DR patients, compared to the NDR patients, and the concentration of only one carnitine, C7:DC, was significantly increased in the PDR patients, compared to the NPDR patients [1]. Furthermore, the concentrations of other carnitines, C14:1 and C16, were decreased in the DR (NPDR and PDR) patients, compared to the NDR patients (Tables 2 and S1–S3). Due to the difference in the analysis platform, the metabolites (except some of the carnitines) that were significantly different in the DR and PDR patients were not the same as those selected in the previous studies. Since our study, unlike previous studies, included many carnitine subtypes, the levels of some carnitines in our study did not correspond to those in previous studies.

There are some limitations in our study. First, we used serum samples instead of vitreous humor samples to analyze the metabolites related to DR. Sampling of the vitreous humor in human subjects is not recommended (for research purposes only); the collection implies an invasive procedure, even more difficult to justify in healthy individuals (the control group). Also, related to this (in the context of approved protocols), it is difficult to recruit healthy humans, especially in large numbers. Hence, using vitreous humor samples to define biomarkers for DR risk prediction is also not without limitations; study replication is quite difficult. In fact, to date, there are only four reports available involving vitreous humor samples for the analysis of metabolites related to DR. This said, we still tried to compare the selected metabolites in our study to those suggested by other studies using vitreous samples [28–31]. Of note, six metabolites including proline, citrulline, kynurenine, creatinine, glutamine, and methionine were common between our study (Tables 2 and S1–S3) and the previous studies. Considering the different sources of samples (serum and vitreous humor), the commonality of these six metabolites may indicate that serum samples can be used for discovery of DR-specific metabolites, even though vitreous humor better represents the status of DR. Serum samples include all kinds of metabolites originated from whole body functions. To discover DR-specific metabolites, we excluded patients having other diabetic complications such as diabetic nephropathy; the samples used were collected from patients with DR only. Therefore, we believe that the DR samples used in this study were appropriate; the fact that the selected metabolites in our study, such as total DMA, tryptophan, kynurenine, creatinine, and carnitine, were also reported as DR-related metabolites in previous studies support our viewpoint.

Second, NPDR could have been sub-categorized into several stages such as ‘mild’, ‘moderate’, and ‘severe’. In fact, we believe that such a sub-characterization would be important to investigate potential differences in metabolites among the three stages, and to better understand the disease. However, due to the lack of stage information with respect to NDPR patients, we could not follow the abovementioned approach in this study.

Third, we have compared DR to NDR patients to identify the metabolites related to the progression of DR in diabetic patients. The enrolment of non-DM patients in the study, as healthy controls (of NDR diabetes patients), would have also been interesting. However, the serum of healthy individuals was not available at the time the study was conducted. Instead, we have used a stringent analysis; the NDR group included patients without any kind of diabetic complications, while the DR group included diabetic patients with DR alone (and without other Diabetes complications). Of note, following this approach, we identified several metabolites related to DR, including a few identified in previous studies. This fact supports validates the choice of NDR samples as control samples.

Fourth, we used a targeted metabolomics approach using a high-throughput platform, the Absolute IDQ^®^ p180 Kit, which can analyze 180 metabolites simultaneously in a very small volume of serum. This platform can be applied for rapid detection of metabolites associated with various diseases. However, several PCs showed significantly different levels in the NDR and DR patients in this study. It was difficult to understand the role of these PCs in DR development because not much information on them has been provided in any of the human metabolite databases. However, the identification of the PCs that were reported as metabolites associated with DR in our study will allow future studies to be conducted to understand the roles of PCs in DR progression in T2D patients. Furthermore, this is a cross-sectional study in which metabolites were measured at only one time point. Additionally, several indicative metabolites for DR progression in T2D patients identified in our study have already been identified in previous reports. These included total DMA, tryptophan, kynurenine, several carnitines, and creatinine, which showed significantly different levels in the DR patients compared to the NDR patients. It could be speculated that high serum creatinine and glucose levels in the DR patients might have contributed to the different concentrations of these metabolites in the DR patients compared to the NDR patients. Further studies, including validation experiments using different samples and functional studies using animal models will be helpful for the identification of other important metabolites related to DR development.

Overall, here we revealed via comprehensive metabolomics profiling using a high-throughput platform, several metabolites associated with DR. These new DR-related metabolites should be considered in the study of the mechanism behind the initiation and progression of DR in T2D patients.

## Supporting information

S1 Table Identification of metabolites associated with diabetic retinopathy. (DOCX)

S2 Table Identification of metabolites associated with non-proliferative diabetic retinopathy. (DOCX)

S3 Table Identification of metabolites associated with proliferative diabetic retinopathy. (DOCX)

S4 Table Identification of metabolites associated with non-proliferative diabetic retinopathy versus proliferative diabetic retinopathy. (DOCX)

S5 Table Differential concentrations of kynurenine in the analysis groups. (DOCX)

S1 Fig The concentration of kynurenine in the different groups. Dot- and box-plots are represented. Graphs were plotted using the R package (Stats, version 3.6.2). The Y-axis indicates the metabolite concentration in micromoles (μM). NDR, non-diabetic retinopathy; NPDR, non-proliferative DR; PDR, proliferative diabetic retinopathy. Two asterisks (**) indicate that ANCOVA p-value is lower than 0.01.
(DOCX)
