# Supplementary material for: Metabolomics profiles associated with diabetic retinopathy in type 2 diabetes patients
Source: PLoS One. 2020 Oct 29;15(10):e0241365. doi: 10.1371/journal.pone.0241365 (PMC7595280; doi:10.1371/journal.pone.0241365)
Supplement: S1 Table — (DOCX) [file pone.0241365.s001.docx]

**S1 Table. Identification of the metabolites associated with diabetic retinopathy.**

| **Metabolites** | **Logistic regression** | | **ANCOVA** | |
| --- | --- | --- | --- | --- |
|  | **Odds Ratio** (95% CI) | ***p-value***  (FDR corrected) | **Fold Change** | ***p-value*** |
| Dodecanoylcarnitine (C12) | 0.72  (0.56-0.91) | 2.50E-02 | 0.89 | 6.61E-03 |
| Tetradecenoylcarnitine (C14:1) | 0.63  (0.48-0.82) | 4.32E-03 | 0.87 | 3.51E-04 |
| Tetradecadienylcarnitine (C14:2) | 0.72  (0.56-0.92) | 2.86E-02 | 0.87 | 7.67E-03 |
| Hexadecanoylcarnitine (C16) | 0.59  (0.45-0.75) | 6.68E-04 | 0.86 | 1.95E-05 |
| Octadecenoylcarnitine (C18:1) | 0.69  (0.53-0.88) | 1.43E-02 | 0.87 | 2.86E-03 |
| Octadecadienylcarnitine (C18:2) | 0.66  (0.51-0.85) | 8.70E-03 | 0.87 | 1.45E-03 |
| Propionylcarnitine (C3) | 1.39  (1.08-1.81) | 2.97E-02 | 1.16 | 9.31E-03 |
| Butyrylcarnitine (C4) | 1.83  (1.33-2.61) | 2.91E-03 | 1.31 | 3.94E-04 |
| Glutamine (Gln) | 0.73  (0.56-0.95) | 4.72E-02 | 1.00 | 2.60E-02 |
| Histidine (His) | 0.69  (0.53-0.88) | 1.33E-02 | 0.95 | 2.77E-03 |
| Lysine (Lys) | 0.63  (0.49-0.81) | 2.90E-03 | 0.92 | 2.15E-04 |
| Methionine (Met) | 0.53  (0.4-0.69) | 1.69E-04 | 0.88 | 9.26E-07 |
| Phenylalanine (Phe) | 0.73  (0.57-0.93) | 3.59E-02 | 0.95 | 1.12E-02 |
| Proline (Pro) | 1.5  (1.09-2.11) | 3.78E-02 | 1.13 | 2.94E-02 |
| Serine (Ser) | 0.74  (0.58-0.94) | 4.26E-02 | 0.93 | 1.81E-02 |
| Threonine (Thr) | 0.69  (0.54-0.88) | 1.60E-02 | 0.91 | 4.14E-03 |
| Tryptophan (Trp) | 0.36  (0.26-0.49) | 4.48E-08 | 0.81 | 1.79E-12 |
| Tyrosine (Tyr) | 0.43  (0.31-0.57) | 1.42E-06 | 0.84 | 4.84E-10 |
| Creatinine | 1.73  (1.22-2.63) | 1.70E-02 | 1.27 | 3.20E-03 |
| Total Dimethyarginine (Total DMA) | 2.3  (1.59-3.47) | 4.28E-04 | 1.31 | 4.73E-05 |
| lysoPhosphatidylcholine acyl C18:0 (lysoPC a C18:0) | 0.73  (0.57-0.93) | 3.30E-02 | 0.90 | 9.85E-03 |
| lysoPhosphatidylcholine acyl C18:2 (lysoPC a C18:2) | 0.7  (0.54-0.9) | 2.50E-02 | 0.85 | 6.76E-03 |
| Phosphatidylcholine diacyl C28:1  (PC aa C28:1) | 0.74  (0.58-0.95) | 4.72E-02 | 0.90 | 1.66E-02 |
| Phosphatidylcholine diacyl C32:0  (PC aa C32:0) | 0.72  (0.56-0.91) | 2.64E-02 | 0.93 | 7.29E-03 |
| Phosphatidylcholine diacyl C32:2  (PC aa C32:2) | 0.47  (0.34-0.62) | 1.82E-05 | 0.75 | 4.79E-08 |
| Phosphatidylcholine diacyl C32:3  (PC aa C32:3) | 0.63  (0.48-0.81) | 2.90E-03 | 0.87 | 2.86E-04 |
| Phosphatidylcholine diacyl C34:2  (PC aa C34:2) | 0.56  (0.42-0.73) | 6.68E-04 | 0.85 | 2.24E-05 |
| Phosphatidylcholine diacyl C34:3  (PC aa C34:3) | 0.74  (0.57-0.94) | 4.26E-02 | 0.91 | 1.69E-02 |
| Phosphatidylcholine diacyl C34:4  (PC aa C34:4) | 0.64  (0.49-0.82) | 3.94E-03 | 0.84 | 4.03E-04 |
| Phosphatidylcholine diacyl C36:0  (PC aa C36:0) | 0.67  (0.51-0.85) | 9.18E-03 | 0.87 | 1.18E-03 |
| Phosphatidylcholine diacyl C36:1  (PC aa C36:1) | 0.75  (0.59-0.95) | 4.92E-02 | 0.92 | 1.85E-02 |
| Phosphatidylcholine diacyl C36:2  (PC aa C36:2) | 0.56  (0.43-0.73) | 4.28E-04 | 0.85 | 1.10E-05 |
| Phosphatidylcholine diacyl C36:6  (PC aa C36:6) | 0.67  (0.52-0.86) | 9.04E-03 | 0.87 | 1.29E-03 |
| Phosphatidylcholine diacyl C38:0  (PC aa C38:0) | 0.72  (0.54-0.93) | 4.60E-02 | 0.89 | 1.24E-02 |
| Phosphatidylcholine diacyl C38:3  (PC aa C38:3) | 0.72  (0.56-0.91) | 2.53E-02 | 0.91 | 6.41E-03 |
| Phosphatidylcholine diacyl C38:5  (PC aa C38:5) | 0.73  (0.57-0.93) | 3.66E-02 | 0.94 | 1.10E-02 |
| Phosphatidylcholine diacyl C38:6  (PC aa C38:6) | 0.61  (0.47-0.78) | 1.49E-03 | 0.86 | 8.06E-05 |
| Phosphatidylcholine diacyl C40:5  (PC aa C40:5) | 0.69  (0.54-0.88) | 1.43E-02 | 0.92 | 3.00E-03 |
| Phosphatidylcholine diacyl C40:6  (PC aa C40:6) | 0.6  (0.46-0.77) | 1.15E-03 | 0.87 | 5.91E-05 |
| Phosphatidylcholine diacyl C42:2  (PC aa C42:2) | 0.66  (0.51-0.84) | 6.59E-03 | 0.86 | 7.11E-04 |
| Phosphatidylcholine diacyl C42:5  (PC aa C42:5) | 0.72  (0.56-0.92) | 2.97E-02 | 0.92 | 9.29E-03 |
| Phosphatidylcholine acyl-alkyl C32:1  (PC ae C32:1) | 0.6  (0.46-0.77) | 8.29E-04 | 0.88 | 3.12E-05 |
| Phosphatidylcholine acyl-alkyl C32:2  (PC ae C32:2) | 0.56  (0.43-0.72) | 3.75E-04 | 0.85 | 3.20E-06 |
| Phosphatidylcholine acyl-alkyl C34:0  (PC ae C34:0) | 0.74  (0.58-0.94) | 4.26E-02 | 0.92 | 1.40E-02 |
| Phosphatidylcholine acyl-alkyl C34:2  (PC ae C34:2) | 0.56  (0.42-0.74) | 8.10E-04 | 0.83 | 4.94E-05 |
| Phosphatidylcholine acyl-alkyl C34:3  (PC ae C34:3) | 0.55  (0.42-0.72) | 3.80E-04 | 0.84 | 6.10E-06 |
| Phosphatidylcholine acyl-alkyl C36:2  (PC ae C36:2) | 0.74  (0.58-0.95) | 4.72E-02 | 0.91 | 1.89E-02 |
| Phosphatidylcholine acyl-alkyl C36:3 (PC ae C36:3) | 0.69  (0.52-0.89) | 1.87E-02 | 0.89 | 4.74E-03 |
| Phosphatidylcholine acyl-alkyl C36:5  (PC ae C36:5) | 0.67  (0.51-0.86) | 1.03E-02 | 0.87 | 1.46E-03 |
| Phosphatidylcholine acyl-alkyl C38:0 (PC ae C38:0) | 0.73  (0.57-0.92) | 2.97E-02 | 0.91 | 7.89E-03 |
| Phosphatidylcholine acyl-alkyl C38:1  (PC ae C38:1) | 0.66  (0.5-0.86) | 1.21E-02 | 0.84 | 1.45E-03 |
| Phosphatidylcholine acyl-alkyl C38:2  (PC ae C38:2) | 0.59  (0.45-0.77) | 1.19E-03 | 0.84 | 4.24E-05 |
| Phosphatidylcholine acyl-alkyl C38:5  (PC ae C38:5) | 0.73  (0.57-0.94) | 4.26E-02 | 0.89 | 1.29E-02 |
| Phosphatidylcholine acyl-alkyl C38:6  (PC ae C38:6) | 0.64  (0.49-0.83) | 4.96E-03 | 0.87 | 4.46E-04 |
| Phosphatidylcholine acyl-alkyl C40:5  (PC ae C40:5) | 0.64  (0.49-0.82) | 3.49E-03 | 0.88 | 2.62E-04 |
| Phosphatidylcholine acyl-alkyl C42:2  (PC ae C42:2) | 0.64  (0.49-0.82) | 3.49E-03 | 0.92 | 3.07E-04 |
| Phosphatidylcholine acyl-alkyl C42:3  (PC ae C42:3) | 0.69  (0.53-0.88) | 1.60E-02 | 0.90 | 2.30E-03 |
| Phosphatidylcholine acyl-alkyl C42:5  (PC ae C42:5) | 0.74  (0.57-0.94) | 4.06E-02 | 0.91 | 1.18E-02 |
| Hydroxysphingomyeline C22:1  (SM (OH) C22:1) | 0.6  (0.46-0.77) | 1.22E-03 | 0.88 | 6.09E-05 |
| Hydroxysphingomyeline C24:1  (SM (OH) C24:1) | 0.67  (0.52-0.86) | 9.04E-03 | 0.91 | 1.25E-03 |
| Sphingomyeline C24:0  (SM C24:0) | 0.57  (0.44-0.74) | 4.28E-04 | 0.88 | 7.84E-06 |
| Hexose (H1) | 1.45  (1.12-1.9) | 1.70E-02 | 1.11 | 3.84E-03 |
