# Supplementary material for: Metabolomics profiles associated with diabetic retinopathy in type 2 diabetes patients
Source: PLoS One. 2020 Oct 29;15(10):e0241365. doi: 10.1371/journal.pone.0241365 (PMC7595280; doi:10.1371/journal.pone.0241365)
Supplement: S2 Table — (DOCX) [file pone.0241365.s002.docx]

**S2 Table. Identification of the metabolites associated with non-proliferative diabetic retinopathy.**

| **Metabolites** | **Logistic regression** | | **ANCOVA** | |
| --- | --- | --- | --- | --- |
|  | **Odds Ratio** (95% CI) | ***p-value***  (FDR corrected) | **Fold Change** | ***p-value*** |
| Carnitine (C0) | 0.73  (0.55-0.95) | 7.64E-02 | 0.96 | 2.15E-02 |
| Tetradecenoylcarnitine (C14:1) | 0.65  (0.48-0.86) | 1.97E-02 | 0.88 | 2.87E-03 |
| Hexadecanoylcarnitine (C16) | 0.62  (0.46-0.81) | 6.11E-03 | 0.87 | 6.24E-04 |
| Alanine (Ala) | 1.31  (1.01-1.73) | 9.11E-02 | 1.05 | 3.83E-02 |
| Arginine (Arg) | 0.76  (0.58-0.99) | 9.27E-02 | 0.97 | 4.00E-02 |
| Asparagine (Asp) | 1.33  (1.02-1.76) | 7.94E-02 | 1.06 | 2.62E-02 |
| Glutamate (Glu) | 1.34  (1.03-1.77) | 7.88E-02 | 1.04 | 2.68E-02 |
| Histidine (His) | 0.66  (0.5-0.87) | 1.83E-02 | 0.95 | 3.19E-03 |
| Lysine (Lys) | 0.65  (0.49-0.86) | 1.57E-02 | 0.92 | 2.16E-03 |
| Methionine (Met) | 0.51  (0.37-0.69) | 6.00E-04 | 0.87 | 9.33E-06 |
| Threonine (Thr) | 0.67  (0.5-0.88) | 2.33E-02 | 0.91 | 5.47E-03 |
| Tryptophan (Trp) | 0.4  (0.28-0.55) | 1.42E-05 | 0.83 | 8.55E-09 |
| Tyrosine (Tyr) | 0.47  (0.34-0.64) | 1.57E-04 | 0.86 | 8.42E-07 |
| Total Dimethyarginine (Total DMA) | 1.61  (1.2-2.21) | 9.41E-03 | 1.21 | 1.08E-03 |
| lysoPhosphatidylcholine acyl C16:0  (lysoPC a C16:0) | 0.75  (0.57-0.98) | 8.44E-02 | 0.91 | 3.92E-02 |
| Phosphatidylcholine diacyl C30:0  (PC aa C30:0) | 0.76  (0.58-0.99) | 9.27E-02 | 0.92 | 4.52E-02 |
| Phosphatidylcholine diacyl C32:2  (PC aa C32:2) | 0.41  (0.29-0.58) | 5.18E-05 | 0.75 | 1.54E-07 |
| Phosphatidylcholine diacyl C32:3  (PC aa C32:3) | 0.59  (0.43-0.78) | 3.69E-03 | 0.86 | 3.22E-04 |
| Phosphatidylcholine diacyl C34:2  (PC aa C34:2) | 0.53  (0.38-0.72) | 1.47E-03 | 0.85 | 5.73E-05 |
| Phosphatidylcholine diacyl C34:4  (PC aa C34:4) | 0.63  (0.47-0.84) | 1.05E-02 | 0.84 | 1.55E-03 |
| Phosphatidylcholine diacyl C36:0  (PC aa C36:0) | 0.58  (0.42-0.77) | 3.69E-03 | 0.86 | 1.74E-04 |
| Phosphatidylcholine diacyl C36:2  (PC aa C36:2) | 0.57  (0.42-0.75) | 2.02E-03 | 0.86 | 7.68E-05 |
| Phosphatidylcholine diacyl C36:6  (PC aa C36:6) | 0.63  (0.47-0.83) | 9.01E-03 | 0.86 | 8.49E-04 |
| Phosphatidylcholine diacyl C38:0  (PC aa C38:0) | 0.59  (0.43-0.79) | 4.30E-03 | 0.87 | 2.89E-04 |
| Phosphatidylcholine diacyl C38:1  (PC aa C38:1) | 0.73  (0.54-0.95) | 7.75E-02 | 0.89 | 2.03E-02 |
| Phosphatidylcholine diacyl C38:6  (PC aa C38:6) | 0.58  (0.43-0.77) | 3.69E-03 | 0.86 | 1.36E-04 |
| Phosphatidylcholine diacyl C40:6  (PC aa C40:6) | 0.63  (0.47-0.82) | 7.30E-03 | 0.89 | 6.75E-04 |
| Phosphatidylcholine diacyl C42:1  (PC aa C42:1) | 0.72  (0.54-0.94) | 6.58E-02 | 0.92 | 1.63E-02 |
| Phosphatidylcholine diacyl C42:2  (PC aa C42:2) | 0.62  (0.46-0.82) | 8.09E-03 | 0.86 | 7.40E-04 |
| Phosphatidylcholine acyl-alkyl C32:1  (PC ae C32:1) | 0.55  (0.41-0.73) | 1.02E-03 | 0.87 | 1.97E-05 |
| Phosphatidylcholine acyl-alkyl C32:2  (PC ae C32:2) | 0.48  (0.34-0.64) | 1.35E-04 | 0.84 | 4.10E-07 |
| Phosphatidylcholine acyl-alkyl C34:0  (PC ae C34:0) | 0.67  (0.5-0.88) | 2.33E-02 | 0.91 | 4.06E-03 |
| Phosphatidylcholine acyl-alkyl C34:1  (PC ae C34:1) | 0.75  (0.57-0.97) | 7.94E-02 | 0.95 | 2.81E-02 |
| Phosphatidylcholine acyl-alkyl C34:2  (PC ae C34:2) | 0.49  (0.35-0.67) | 3.86E-04 | 0.82 | 1.01E-05 |
| Phosphatidylcholine acyl-alkyl C34:3  (PC ae C34:3) | 0.5  (0.36-0.67) | 2.25E-04 | 0.83 | 2.11E-06 |
| Phosphatidylcholine acyl-alkyl C36:2  (PC ae C36:2) | 0.67  (0.5-0.88) | 2.08E-02 | 0.90 | 4.31E-03 |
| Phosphatidylcholine acyl-alkyl C36:3  (PC ae C36:3) | 0.61  (0.45-0.81) | 7.04E-03 | 0.87 | 8.65E-04 |
| Phosphatidylcholine acyl-alkyl C36:5  (PC ae C36:5) | 0.67  (0.5-0.89) | 2.62E-02 | 0.88 | 5.26E-03 |
| Phosphatidylcholine acyl-alkyl C38:0  (PC ae C38:0) | 0.68  (0.51-0.89) | 2.79E-02 | 0.90 | 4.67E-03 |
| Phosphatidylcholine acyl-alkyl C38:1  (PC ae C38:1) | 0.63  (0.45-0.84) | 1.83E-02 | 0.83 | 2.03E-03 |
| Phosphatidylcholine acyl-alkyl C38:2  (PC ae C38:2) | 0.56  (0.41-0.75) | 3.13E-03 | 0.84 | 7.56E-05 |
| Phosphatidylcholine acyl-alkyl C38:6  (PC ae C38:6) | 0.58  (0.42-0.77) | 3.69E-03 | 0.86 | 1.55E-04 |
| Phosphatidylcholine acyl-alkyl C40:1  (PC ae C40:1) | 0.75  (0.57-0.97) | 7.97E-02 | 0.90 | 2.60E-02 |
| Phosphatidylcholine acyl-alkyl C40:2  (PC ae C40:2) | 0.72  (0.54-0.95) | 6.77E-02 | 0.94 | 2.13E-02 |
| Phosphatidylcholine acyl-alkyl C40:3  (PC ae C40:3) | 0.74  (0.56-0.97) | 8.28E-02 | 0.94 | 3.16E-02 |
| Phosphatidylcholine acyl-alkyl C40:5  (PC ae C40:5) | 0.61  (0.45-0.81) | 7.04E-03 | 0.88 | 5.21E-04 |
| Phosphatidylcholine acyl-alkyl C40:6  (PC ae C40:6) | 0.75  (0.57-0.97) | 8.35E-02 | 0.93 | 3.13E-02 |
| Phosphatidylcholine acyl-alkyl C42:2  (PC ae C42:2) | 0.59  (0.44-0.78) | 3.69E-03 | 0.91 | 1.63E-04 |
| Phosphatidylcholine acyl-alkyl C42:3  (PC ae C42:3) | 0.68  (0.5-0.89) | 3.24E-02 | 0.91 | 4.66E-03 |
| Hydroxysphingomyeline C22:1  (SM (OH) C22:1) | 0.6  (0.44-0.8) | 5.02E-03 | 0.88 | 4.87E-04 |
| Hydroxysphingomyeline C24:1  (SM (OH) C24:1) | 0.66  (0.49-0.86) | 1.68E-02 | 0.91 | 2.61E-03 |
| Sphingomyeline C24:0  (SM C24:0) | 0.6  (0.45-0.8) | 4.30E-03 | 0.89 | 2.89E-04 |
| Hexose (H1) | 1.43  (1.09-1.91) | 3.47E-02 | 1.09 | 8.28E-03 |
