# Supplementary material for: Metabolomics profiles associated with diabetic retinopathy in type 2 diabetes patients
Source: PLoS One. 2020 Oct 29;15(10):e0241365. doi: 10.1371/journal.pone.0241365 (PMC7595280; doi:10.1371/journal.pone.0241365)
Supplement: S3 Table — (DOCX) [file pone.0241365.s003.docx]

**S3 Table. Identification of the metabolites associated with proliferative diabetic retinopathy.**

| **Metabolites** | **Logistic regression** | | **ANCOVA** | |
| --- | --- | --- | --- | --- |
|  | **Odds Ratio** (95% CI) | ***p-value***  (FDR corrected) | **Fold Change** | ***p-value*** |
| Tetradecenoylcarnitine (C14:1) | 0.53  (0.33-0.8) | 3.01E-02 | 0.84 | 4.21E-03 |
| Hexadecanoylcarnitine (C16) | 0.47  (0.3-0.71) | 7.83E-03 | 0.82 | 3.13E-04 |
| Octadecanoylcarnitine (C18) | 0.56  (0.36-0.84) | 3.90E-02 | 0.83 | 1.37E-02 |
| Octadecenoylcarnitine (C18:1) | 0.52  (0.33-0.77) | 1.94E-02 | 0.81 | 1.98E-03 |
| Octadecadienylcarnitine (C18:2) | 0.47  (0.3-0.7) | 7.83E-03 | 0.81 | 6.21E-04 |
| Propionylcarnitine (C3) | 1.85  (1.3-2.67) | 8.96E-03 | 1.25 | 3.08E-04 |
| Butyrylcarnitine (C4) | 3.88  (2.35-6.95) | 3.92E-05 | 1.63 | 1.69E-10 |
| Valerycarnitine (C5) | 1.58  (1.15-2.22) | 3.24E-02 | 1.23 | 2.86E-03 |
| Citrulline (Cit) | 1.66  (1.18-2.4) | 3.01E-02 | 1.31 | 1.38E-03 |
| Lysine (Lys) | 0.6  (0.41-0.85) | 3.44E-02 | 0.92 | 5.51E-03 |
| Methionine (Met) | 0.51  (0.32-0.77) | 2.32E-02 | 0.88 | 2.01E-03 |
| Serine (Ser) | 0.54  (0.36-0.79) | 1.94E-02 | 0.89 | 1.91E-03 |
| Tryptophan (Trp) | 0.25  (0.14-0.4) | 2.81E-05 | 0.78 | 6.31E-09 |
| Tyrosine (Tyr) | 0.26  (0.14-0.43) | 3.92E-05 | 0.79 | 7.64E-08 |
| Creatinine | 3.46  (1.93-7.13) | 5.92E-03 | 1.63 | 8.79E-08 |
| Kynurenine | 1.75  (1.21-2.61) | 3.01E-02 | 1.26 | 1.13E-03 |
| Total Dimethyarginine (Total DMA) | 4.45  (2.48-8.72) | 9.85E-05 | 1.56 | 1.27E-08 |
| lysoPhosphatidylcholine acyl C18:2 (lysoPC a C18:2) | 0.53  (0.33-0.81) | 3.01E-02 | 0.81 | 6.92E-03 |
| Phosphatidylcholine diacyl C32:2  (PC aa C32:2) | 0.47  (0.3-0.72) | 9.68E-03 | 0.75 | 8.41E-04 |
| Phosphatidylcholine diacylC34:2  (PC aa C34:2) | 0.52  (0.33-0.79) | 3.01E-02 | 0.85 | 5.15E-03 |
| Phosphatidylcholine diacyl C36:2  (PC aa C36:2) | 0.47  (0.3-0.7) | 7.83E-03 | 0.83 | 3.97E-04 |
| Phosphatidylcholine diacyl C38:3  (PC aa C38:3) | 0.6  (0.4-0.86) | 4.09E-02 | 0.88 | 6.87E-03 |
| Phosphatidylcholine diacyl C38:6  (PC aa C38:6) | 0.59  (0.39-0.86) | 4.09E-02 | 0.86 | 5.50E-03 |
| Phosphatidylcholine diacyl C40:5  (PC aa C40:5) | 0.59  (0.39-0.86) | 4.78E-02 | 0.88 | 5.18E-03 |
| Phosphatidylcholine diacyl C40:6  (PC aa C40:6) | 0.49  (0.32-0.72) | 8.83E-03 | 0.83 | 1.70E-04 |
| Phosphatidylcholine diacyl C42:5  (PC aa C42:5) | 0.46  (0.28-0.71) | 9.81E-03 | 0.83 | 5.03E-04 |
| Phosphatidylcholine acyl-alkyl C36:5  (PC ae C36:5) | 0.57  (0.37-0.85) | 4.09E-02 | 0.86 | 1.09E-02 |
| Phosphatidylcholine acyl-alkyl C42:3  (PC ae C42:3) | 0.57  (0.38-0.83) | 3.01E-02 | 0.88 | 2.97E-03 |
| Hydroxysphingomyeline C22:1  (SM (OH) C22:1) | 0.56  (0.36-0.82) | 3.01E-02 | 0.88 | 5.35E-03 |
| Sphingomyeline C24:0  (SM C24:0) | 0.47  (0.31-0.7) | 7.45E-03 | 0.87 | 1.72E-04 |
