# Supplementary material for: Metabolomics profiles associated with diabetic retinopathy in type 2 diabetes patients
Source: PLoS One. 2020 Oct 29;15(10):e0241365. doi: 10.1371/journal.pone.0241365 (PMC7595280; doi:10.1371/journal.pone.0241365)
Supplement: S4 Table — (DOCX) [file pone.0241365.s004.docx]

**S4 Table. The metabolites associated with non-proliferative diabetic retinopathy versus proliferative diabetic retinopathy.**

| **Metabolites** | **Logistic regression** | | **ANCOVA** | |
| --- | --- | --- | --- | --- |
|  | **Odds Ratio** (95% CI) | ***p-value***  (FDR corrected) | **Fold Change** | ***p-value*** |
| Pimelylcartinine (C7:DC) | 1.76  (1.05-3.18) | 4.00E-02 | 1.10 | 1.70E-01 |
| Creatinine | 2.22  (1.33-4.25) | 6.00E-03 | 1.44 | 2.23E-05 |
| Total Dimethyarginine (Total DMA) | 1.64  (1.14-2.58) | 2.00E-02 | 1.30 | 4.03E-04 |
| lysoPhosphatidylcholine acyl C18:2 (lysoPC a C18:2) | 0.63  (0.39-0.96) | 4.00E-02 | 0.93 | 3.47E-01 |
| Phosphatidylcholine diacyl C32:2  (PC aa C32:2) | 2.1  (1.21-4.1) | 2.00E-02 | 1.01 | 9.64E-01 |
| Phosphatidylcholine diacyl C36:1  (PC aa C36:1) | 0.61  (0.38-0.93) | 3.00E-02 | 0.97 | 5.21E-01 |
| Phosphatidylcholine acyl-alkyl C42:3  (PC ae C44:4) | 0.68  (0.44-0.97) | 5.00E-02 | 0.99 | 5.87E-01 |
| Phosphatidylcholine acyl-alkyl C42:3  (PC ae C44:5) | 0.56  (0.34-0.88) | 2.00E-02 | 0.95 | 3.43E-01 |
