# Supplementary material for: Metabolomics profiles associated with diabetic retinopathy in type 2 diabetes patients
Source: PLoS One. 2020 Oct 29;15(10):e0241365. doi: 10.1371/journal.pone.0241365 (PMC7595280; doi:10.1371/journal.pone.0241365)
Supplement: S5 Table — (DOCX) [file pone.0241365.s005.docx]

**S5 Table. The concentration of kynurenine in the different groups; statistical analysis.**

| **Analysis Group** | **Logistic regression** | | **ANCOVA** | |
| --- | --- | --- | --- | --- |
|  | **Odds Ratio** (95% CI) | ***p-value***  (FDR corrected) | **Fold Change** | ***p-value*** |
| DR vs NDR | 1.29  (0.99–1.72) | 7.00E-01 | 1.15 | 7.E-02 |
| NPDR vs NDR | 1.14  (0.86–1.51) | 4.00E-01 | 1.12 | 3.E-01 |
| PDR vs NDR | 1.75  (1.21–2.61) | 3.00E-02 | 1.26 | 1.E-03 |
| PDR vs NPDR | 1.32  (0.97–1.84) | 8.00E-02 | 1.12 | 7.E-02 |

* DR, diabetic retinopathy; NDR, non-diabetic retinopathy; NPDR, non-proliferative diabetic retinopathy; PDR, proliferative diabetic retinopathy
