# Supplementary material for: Metabolomics profiles associated with diabetic retinopathy in type 2 diabetes patients
Source: PLoS One. 2020 Oct 29;15(10):e0241365. doi: 10.1371/journal.pone.0241365 (PMC7595280; doi:10.1371/journal.pone.0241365)

**S1 Figure. The concentration of kynurenine in the different groups.** Dot- and box-plots are represented. Graphs were plotted using the R package (Stats, version 3.6.2). The Y-axis indicates the metabolite concentration in microMoles (µM). NDR, non-diabetic retinopathy; NPDR, non-proliferative DR; PDR, proliferative diabetic retinopathy. Two asterisks (**) indicate that ANCOVA p-value is lower than 0.01.


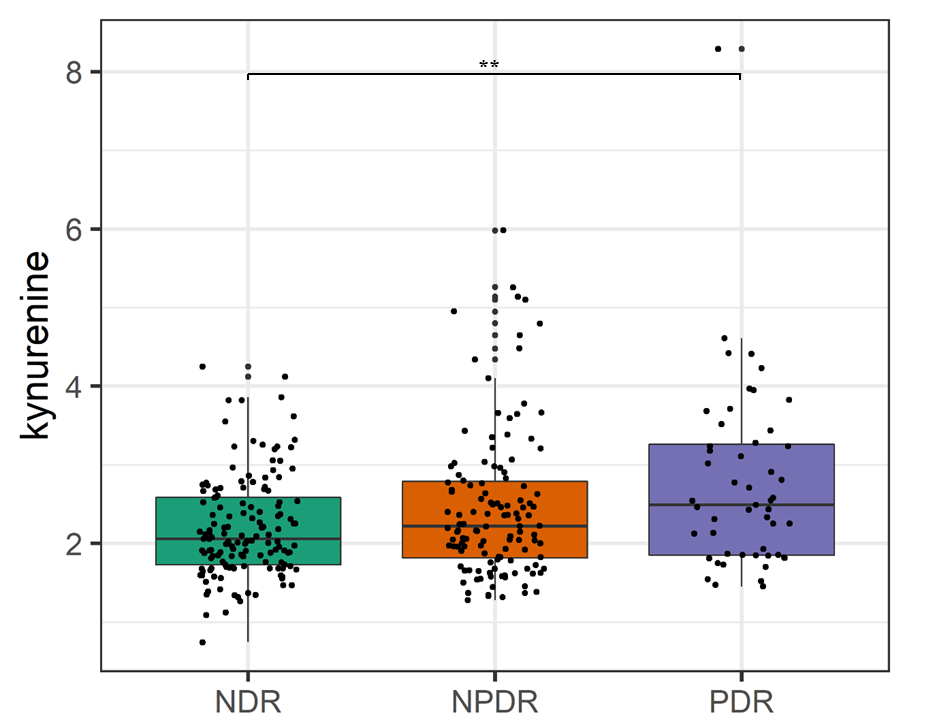

Supplement: S1 Fig — Dot- and box-plots are represented. Graphs were plotted using the R package (Stats, version 3.6.2). The Y-axis indicates the metabolite concentration in micromoles (μM). NDR, non-diabetic retinopathy; NPDR, non-proliferative DR; PDR, proliferative diabetic retinopathy. Two asterisks (**) indicate that ANCOVA p-value is lower than 0.01. (DOCX) [file pone.0241365.s006.docx]
